# Community Composition, Assembly Processes and Stability of Microeukaryotic Plankton in Response to Damming-Altered Heterogeneous Hydrology in a Sediment-Laden River

**DOI:** 10.3390/biology15120945

**Published:** 2026-06-17

**Authors:** Huatao Yuan, Junjun Mei, Xucong Lyu, Xiaofei Gao, Jing Dong, Jingxiao Zhang, Penghui Zhu, Yunni Gao, Xuejun Li

**Affiliations:** 1 College of Fisheries, Henan Normal University, Xinxiang 453007, China; yuanhuatao@htu.edu.cn (H.Y.);; 2 Observation and Research Station on Water Ecosystem in Danjiangkou Reservoir of Henan Province, Nanyang 474450, China; 3 The National Ecological Quality Comprehensive Monitoring Station (Hebi Station), Hebi 458000, China

**Keywords:** microeukaryotic plankton, environmental DNA, community stability, community assembly, sediment-laden river

## Abstract

Suspended particulate matter (SPM) is a key environmental driver in aquatic ecosystems and plays a significant role in shaping microbial communities, particularly in sediment-rich rivers. Dam construction alters hydrological regimes and creates distinct SPM gradients; however, the response mechanisms of microeukaryotic plankton communities remain poorly understood. In this study, we used 18S rRNA gene high-throughput sequencing to characterize microeukaryotic plankton communities across riverine, lacustrine, and transitional zones of the Xiaolangdi Reservoir on the Yellow River (China). Our results revealed distinct community compositions in the lacustrine zone, with SPM identified as the primary factor driving community differentiation. Alpha diversity was highest in the riverine zone, while beta diversity differences among zones were dominated by species turnover. Dominant taxa included Cryptophyta (44.71% ± 30.79%), Metazoa (18.98% ± 17.71%), Perkinsea (7.97% ± 9.78%), Chlorophyta (7.06% ± 5.80%), and Dinophyta (6.06% ± 6.73%). Metazoa, Dinophyta, and Phaeophyta were enriched in high-SPM riverine waters, whereas Alveolata dominated low-SPM lacustrine zones. Community assembly was primarily deterministic, governed mainly by homogeneous selection, with stochastic processes exerting stronger influence in riverine zones. Network analysis indicated that riverine zones exhibited more complex and stable networks, lacustrine zones showed higher local but lower global connectivity, and transitional zones displayed stronger interactions but lower stability. These findings advance our understanding of microeukaryotic plankton responses to dam-induced environmental changes and provide a basis for assessing biodiversity impacts in regulated river systems.

## Simple Summary

Dams change how rivers flow and affect suspended particulate matter (SPM), which is a key factor shaping tiny aquatic organisms called microeukaryotic plankton community. In the Xiaolangdi Reservoir of China’s Yellow River, we found that SPM mainly drove community differences across riverine, transitional, and lacustrine zones. The riverine zone had the highest species diversity and more complex, stable ecological networks. Cryptophyta were most abundant overall, but Metazoa and Dinophyta thrived in high-SPM river waters, while Alveolata dominated low-SPM lake-like zones. Community assembly was mostly driven by deterministic processes (especially homogeneous selection), though random processes played a stronger role in riverine zones. These insights help predict dam impacts on river biodiversity.

## 1. Introduction

The reservoir formed by dam construction, a semi-natural and semi-artificial ecosystem situated between rivers and lakes, possesses unique morphological structures and hydrodynamic characteristics that induce heterogeneities in the river hydrological environment, as well as changes in sediment conditions, wetland morphology and geomorphology [1]. The longitudinal profile of the type of reservoir basin presents a triangular shape, with an obvious gradient classification from the reservoir entrance to the dam, where the deepest part is generally located. Along the longitudinal gradient of the reservoir, it can be divided into three zones: the riverine zone (RZ), the transition zone (TZ), and the lacustrine zone (LZ) [2]. Dam construction reduces the longitudinal, lateral, and vertical connectivity of rivers, alters river sediment transport, fluvial geomorphology, as well as the natural hydrological and thermal regimes of rivers. These habitat changes significantly affect the species richness and community structure of aquatic organisms, such as salmon, the “Four Major Chinese Carps” (black carp, grass carp, silver carp, bighead carp), sturgeons, and eels [3,4]. Particularly, the alteration of hydrological processes, lead to a significant narrowing of the spawning window and population decline of *Schizothorax lissolabiatus* [5]. Eukaryotic plankton, serving as key indicators of aquatic environmental changes, play essential roles in maintaining ecosystem stability, biogeochemical cycles, and energy flow within reservoir ecosystems [6]. However, the impact of dam construction on the diversity of eukaryotic plankton in rivers remains unclear.

In aquatic ecosystems, eukaryotic plankton is characterized by diverse morphologies, rich species diversity, wide distribution, and complex trophic types [7]. Based on diameter, plankton are typically classified into three categories: microplankton (20–200 μm), nanoplankton (3–20 μm), and picoplankton (0.2–3 μm), following the widely cited scheme of Sieburth et al. (1978) [8]. Among these, microeukaryotic plankton refers to taxa with particle sizes less than 200 μm, encompassing fungi, eukaryotic algae, protists, and small metazoans, which are recognized as the most abundant eukaryotic group on Earth [9]. Microeukaryotic plankton demonstrates high sensitivity to environmental changes. The dynamic changes in microeukaryotic plankton not only exert profound impacts on energy flow and biogeochemical cycles but also further alter ecosystem functions [10]. At both the population and community levels, it responds to environmental disturbances and stressors, which in turn result in distinct distribution patterns across different environments [11]. For example, water temperature, salinity, and pH influence the community composition of microeukaryotic plankton through various mechanisms. Consequently, characterizing the diversity, distribution patterns, and correlations with physicochemical factors of microeukaryotic plankton is an essential first step toward understanding the structure, functions, and operational mechanisms of ecosystems.

The mechanisms underlying the composition and geographical distribution patterns of microbial communities in aquatic ecosystems are critical for understanding biogeochemical cycles and ecosystem functions, and also represent a core topic in contemporary community ecology [12]. Community assembly mechanisms primarily consist of deterministic processes and stochastic processes. Based on niche theory, deterministic processes emphasize the role of environmental filtering (e.g., temperature, humidity) and interspecific interactions (e.g., competition) in shaping community structure [13]. In contrast, stochastic processes are rooted in neutral theory, asserting that community structure is mainly determined by random events such as reproduction, mortality, dispersal, and genetic drift [14], with a focus on the effects of ecological drift and probabilistic dispersal [15]. In practice, these two types of processes often jointly influence community assembly [16]. In the heavily sediment-laden Yellow river, the influence of homogeneous selection (stochastic process) on phytoplankton community assembly is enhanced in areas with high sediment load, while the effect of dispersal limitation (deterministic process) is reduced [17].

Furthermore, microorganisms do not exist in isolation; instead, they are interconnected through complex interspecific interaction networks, collectively maintaining ecological functions [18]. Thus, deciphering changes in microbial network structure has become an essential component of microbial ecology [19,20]. Currently, network analysis has emerged as a key tool for investigating microbial interactions [21,22], enabling the revelation of complex interrelationships within communities [23]. By means of topological characteristics—including node attributes, average path length, clustering coefficient, and modularity—network analysis can infer ecological relationships, assess community stability and robustness, and deepen the understanding of how communities respond to natural and anthropogenic disturbances [24].

In the early stages, research on microeukaryotic plankton often employed methods developed for different research objectives. Traditional morphological identification via microscopy, for instance, has been fundamental for characterizing larger microeukaryotes, but it struggles to resolve the diversity of smaller size fractions (e.g., nano- and picoplankton) and cryptic species [25]. Flow cytometry has proven particularly effective in quantifying and sorting these smaller planktonic cells, providing high-resolution data on their physiological states and abundance, yet it typically offers limited taxonomic information [26]. At broader scales, satellite remote sensing provides invaluable insights into phytoplankton biomass (e.g., chlorophyll-*a* concentration), primary productivity, and large-scale spatiotemporal dynamics, albeit with very coarse taxonomic resolution [27]. Chromatographic analysis, while useful for characterizing specific biochemical markers (e.g., pigments), also does not directly address taxonomic identity [28]. Consequently, despite their respective strengths, these approaches individually cannot comprehensively capture the full taxonomic diversity of microeukaryotic plankton [29]. In recent years, with the continuous maturation of high-throughput sequencing (HTS) and DNA metabarcoding technologies, these techniques have become indispensable for biodiversity research. Their high sequencing throughput, capacity to detect rare or novel taxa, and time efficiency enable a more complete and discovery-oriented assessment of microeukaryotic community composition, complementing the methods described above. [30,31]. For instance, using 18S rDNA amplicon sequencing technology found that algal bloom event significantly altered the community composition and the diversity of rare eukaryotic plankton, but had no impact on the diversity of abundant eukaryotic plankton [32]. Gran-Stadniczenko et al. observed a strong seasonal variation in the composition and proportional abundance of protists (unicellular eukaryotes) in the Skagerrak Sea, with temperature being the primary driver of changes in species diversity [33]. Mo et al. demonstrated that a slight increase in salinity exerted selective pressure on microeukaryotic plankton, thereby reducing the diversity of microeukaryotic plankton and altering the community assembly mechanisms as well as network stability [34]. Yang et al. analyzed the spatiotemporal dynamics and assembly processes of microbial communities in the middle reaches of the Yarlung Zangbo River [19].

As the second longest river in China, the Yellow River is knowned for its high sediment load and elevated concentration of suspended particulate matter [35]. Previous investigations on the Yellow River’s aquatic biota have mainly concentrated on the bacterial community [36,37,38]. However, knowledge regarding the diversity and community structure of microeukaryotic plankton—which includes protists, fungi, and small metazoans—remains extremely limited, particularly using modern molecular techniques such as DNA metabarcoding [39]. Therefore, what is currently known about the Yellow River is largely confined to its physical and chemical properties and a few specific taxonomic groups, leaving a significant knowledge gap concerning its microeukaryotic plankton diversity and their ecological roles.

In recent decades, a cascade of large dams has been built along the mainstream of the Yellow River, which has made it one of the most manipulated fluvial systems in the world. More than most other dams, Xiaolangdi Dam has been assigned many important tasks due to its key location and large watershed control area [40]. After the impoundment of the Xiaolangdi Reservoir on the Yellow River, the physicochemical properties of water in local river sections have changed (e.g., increased pH value and decreased suspended sediment content), forming a new aquatic environment. In the background, the distribution patterns of microeukaryotic plankton communities and their relationships with environmental factors remain unclear. Currently, research on this reservoir mostly focuses on planktonic bacteria, while the response mechanisms and community assembly processes of microeukaryotic plankton are insufficiently understood. To address this gap, this study intends to analyze the driving factors and interaction patterns of microeukaryotic plankton community in the Xiaolangdi Reservoir based on 18S rDNA amplicon high-throughput sequencing and environmental factor determination, thereby revealing the stability mechanism of this special ecosystem. The following scientific questions will be focused on: (1) The structural characteristics of microeukaryotic plankton community in different water areas with hydrological features (the riverine zone: RZ, the transition zone: TZ, and the lacustrine zone: LZ); (2) The community assembly mechanisms of microeukaryotic plankton community in the aforementioned water areas and the role of environmental factors in their assembly processes; (3) The network co-occurrence patterns of microeukaryotic plankton in different water areas.

## 2. Materials and Methods

### 2.1. Study Area, Sample Collection, and Environmental Measurements

**Figure 1:**
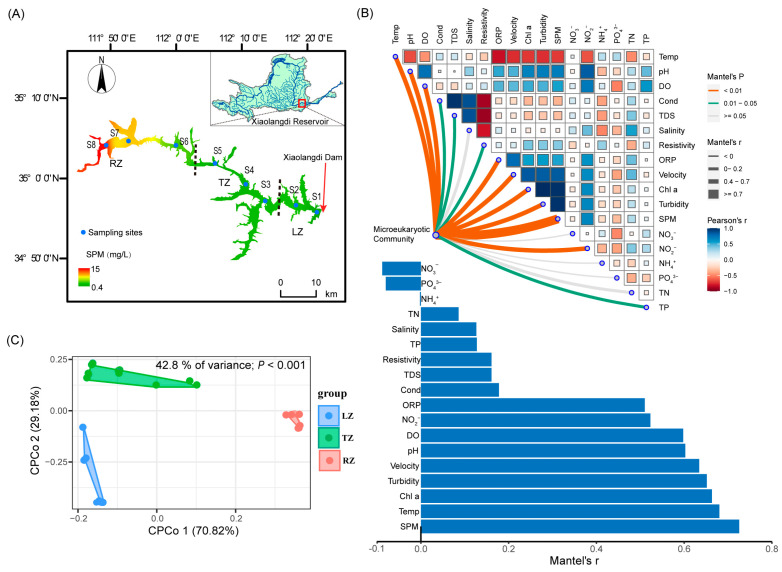
Sampling Stations and Key Drivers of Differentiation in Microeukaryotic Plankton Community in the Xiaolangdi Reservoir. (**A**) Geographical locations of sampling sites, with colors indicating the content of suspended particulate matter; (**B**) Drivers of the microeukaryotic plankton analyzed by the Mantel test, the size of the squares represents the absolute value of the correlation coefficient and larger squares indicate stronger correlations.; (**C**) Differences in community structure among the different zones as demonstrated by CPCoA.

Xiaolangdi Reservoir is a key water conservancy project on the Yellow River, which is located at the outlet of the last gorge in the middle reaches. Based on the distinct hydrological and morphological characteristics of the reservoir, eight sampling sections were established along the main reservoir axis (Figure 1A). Transects S1–S8 are ordered by increasing distance from the dam. Clarification of the legends RZ, TZ, and LZ: (1) LZ (Lacustrine Zone): corresponding to Sections S1–S2, which are under persistent reservoir influence and maintain perennial lacustrine conditions; (2) TZ (Transition Zone): corresponding to Sections S3–S5, which exhibit fluvial characteristics at the minimum water level (230 m) and lacustrine conditions at the maximum operating level (254 m); (3) RZ (Riverine Zone): corresponding to Sections S6–S8, which are unaffected by impoundment and retain year-round fluvial conditions. The total of 24 surface water samples (three replicate samples for each of eight sections) were collected on 18 May 2023. Sampling involved two main components: collection for amplicon sequencing and measurement of water quality factors. Surface water (0.5 m depth) was obtained using a 5L Niskin bottle (Model 1010, General Oceanics, Miami, FL, USA) with volumes of 10~20 L per sample. According to the size classification of Sieburth et al. [8], microplankton is defined as 20~200 μm, nanoplankton as 3~20 μm, and picoplankton as 0.2~3 μm. In this study, the pre-filtration with a 200-μm mesh removed not only large zooplankton and debris but also the fraction of microplankton larger than 200 μm (i.e., the upper part of the microplankton size class, if following alternative classifications that extend microplankton to >200 μm; e.g., some authors define microplankton up to 500 μm). Therefore, our analyzed size fraction (0.2–200 μm) covers the entire picoplankton, nanoplankton, and a substantial portion of typical microplankton (20–200 μm). Prior to filtration, the water was pre-filtered through a 200-μm mesh to remove large plankton. Subsequently, it was passed through a 0.2-μm pore-size 142 mm polycarbonate filter membrane (Cat. No. 112106, Whatman, Cytiva, UK) using a peristaltic pump. After filtration, the membranes were immediately preserved in liquid nitrogen for subsequent DNA extraction.

Physicochemical parameters including water temperature, pH, and salinity at each sampling site were measured using a YSI Professional Plus instrument (YSI Inc., Yellow Springs, OH, USA). Water samples were immediately filtered through 0.2 μm polycarbonate membranes (Millipore, Burlington, MA, USA) after collection to remove particulate matter. The filtrates were used for all subsequent nutrient analyses. Total nitrogen (TN), ammonium nitrogen (NH_4_^+^-N), and nitrate nitrogen (NO_3_^−^-N) were determined via alkaline potassium persulfate digestion-UV spectrophotometry, Nessler’s reagent spectrophotometry, and UV spectrophotometry, respectively. Total phosphorus (TP) and soluble reactive phosphorus (PO_4_^3−^-P) were quantified using the ammonium molybdate spectrophotometric method. The Chlorophyll *a* (Chl *a*) extracted in acetone was analyzed using a Trilogy fluorometer (Turner Design, San Jose, CA, USA) [41].

### 2.2. DNA Extraction, Sequencing, and Data Processing

DNA was extracted using a modified CTAB method [42]. The V4 region of microeukaryotic 18S rRNA gene was amplified via polymerase chain reaction (PCR) with specific primers (18S V4-F: 5′-GGCAAGTCTGGTGCCAG-3′; 18S V4-R: 5′-GACTACGACGGTATCTRATCRTCTTCG-3′) [43]. According to the instructions for paired-end sequencing, amplicon libraries were prepared and sequencing was performed on an Illumina HiSeq 2000 platform (Illumina Inc., San Diego, CA, USA) by Shanghai Majorbio Biomedicine Technology Co., Ltd. (Shanghai, China).

Raw sequencing sequences were subjected to quality control and dereplication. High-quality sequences were obtained by deduplication and chimera removal using the DADA2 plugin in QIIME 2 [44], followed by generation of amplicon sequence variants (ASVs). ASV annotation was performed in R software using the SILVA database (Version 138.1) [45]. For ensuring data accuracy and comparability, low-quality sequence ASVs with low abundance (<10 reads) and low confidence (<0.8) were eliminated prior to data normalization for downstream analysis.

### 2.3. Bioinformatic and Statistical Analysis

The R package “vegan” and “picante” were applied to measure α-diversity (Shannon, Chao1, PD, and Pielou evenness indexs) of each sample. Based on Bray–Curtis distances, constrained Principal Coordinate Analysis (CPCoA) and permutational multivariate analysis of variance (PERMANOVA) were conducted using the “ape” package to evaluate differences in species composition of the microeukaryotic community. The “linkET” package was used to calculate correlations between the microeukaryotic plankton community and environmental factors.

The neutral community model (NCM) was used to determine the potential contribution of neutral (stochastic) processes to the microeukaryotic community at different areas [46]. Additionally, The R package “NST” was employed to compute the phylogenetic normalized stochasticity ratio [47]. The R package “EcolUtils” was used to simulate 1000 permutations for calculating ASV occurrence frequency, and further to compute Levins’ niche breadth, significance testing of niche breadth for each ASV was also conducted. Finally, the framework of phylogenetic bin-based null model analysis (iCAMP) was used to quantitatively infer the contribution of various ecological processes to community assembly in different zones of the reservoir [48], with calculations conducted using the “iCAMP” package.

Co-occurrence network analysis and natural connectivity calculation were performed using the R packages “psych” and “igraph”, and visualization was achieved using Gephi (Version 0.9.2). The R package “microeco” was used for keystone species analysis. Only correlations with statistical significance (*p* < 0.05) and strong correlation strength (Spearman |r| ≥ 0.60) were included. Nodes were classified into four categories based on within-module connectivity (Zi) and among-module connectivity (Pi) of individual nodes: Peripherals (Zi < 2.5, Pi < 0.62), Connectors (Zi < 2.5, Pi > 0.62), Module hubs (Zi > 2.5, Pi < 0.62), and Network hubs (Zi > 2.5, Pi > 0.62), providing a reliable basis for community stability assessment.

Data visualization is performed by the “ggplot2” and the “ggpubr” packages, and partial figure color matching is provided by the “RColorBrewer” package. R^2^ and *p* values are displayed in the figure, using the “ggpmisc” package. All statistical analyses and visualizations were performed in R v4.4.3 (http://www.r-project.org (accessed on 1 February 2026)).

## 3. Results

### 3.1. SPM-Driven Differentiation of Microeukaryotic Plankton Community in the Xiaolangdi Reservoir

Dam construction has induced changes in the hydrological and physicochemical characteristics of three distinct types of water bodies in the Xiaolangdi Reservoir (Table S1). Significant differences were observed among the three zones of the Xiaolangdi Reservoir in pH, conductivity (Cond), dissolved oxygen (DO), flow velocity, Chl *a*, turbidity, suspended particulate matter (SPM), and nitrite nitrogen (NO_2_^−^) (*p* < 0.05). Total dissolved solids (TDS), salinity (Sal), ammonium nitrogen (NH_4_^+^), and orthophosphate (PO_4_^3−^), differed significantly between LZ samples and the other samples (*p* < 0.05), whereas no significant differences were observed between RZ and TZ samples. Along the mainstream of the Yellow River toward the Xiaolangdi Reservoir dam, SPM content varied from 0.4 to 15 mg/L, displaying an overall decreasing trend (Figure 1A). Mantel test analysis demonstrated that SPM, water temperature, turbidity, flow velocity, and other environmental parameters were significantly correlated with microeukaryotic plankton community structure, among which SPM concentration exhibited the strongest association (Figure 1B).

We collected a total of 24 samples from the Xiaolangdi Reservoir and performed total DNA extraction. In each sample, we amplified and sequenced the V4 region of the 18S rRNA gene. Subsequently, we processed the data, which included quality filtering, chimera removal, and rarefaction. We retained 40,246 merged sequences per sample, which were clustered into 4380 ASVs. Based on the Bray–Curtis dissimilarity matrix calculated from the samples, a Constrained Principal Coordinate Analysis (CPCoA) was performed to examine the differences in community composition among different zones in Xiaolangdi Reservoir. The CPCoA analysis revealed that SPM content significantly shaped the microeukaryotic plankton community, explaining 42.8% of the variation (Figure 1C, *p* < 0.01). LZ samples were distinctly separated from others along the primary axis (explaining 70.82% of the constrained variation), while RZ and TZ samples were separated along the secondary axis (explaining the remaining 29.18%). In addition, it is worth noting that α-diversity of the microeukaryotic plankton community decreased significantly with decreasing SPM content. Furthermore, both the Chao1 species richness index and the observed number of species (Observed_species) were significantly positively correlated with SPM content (Figure S2).

### 3.2. Shifts in Community Composition and Diversity of Microeukaryotic Plankton Amomg Different Zones of the Xiaolangdi Reservoir

**Figure 2:**
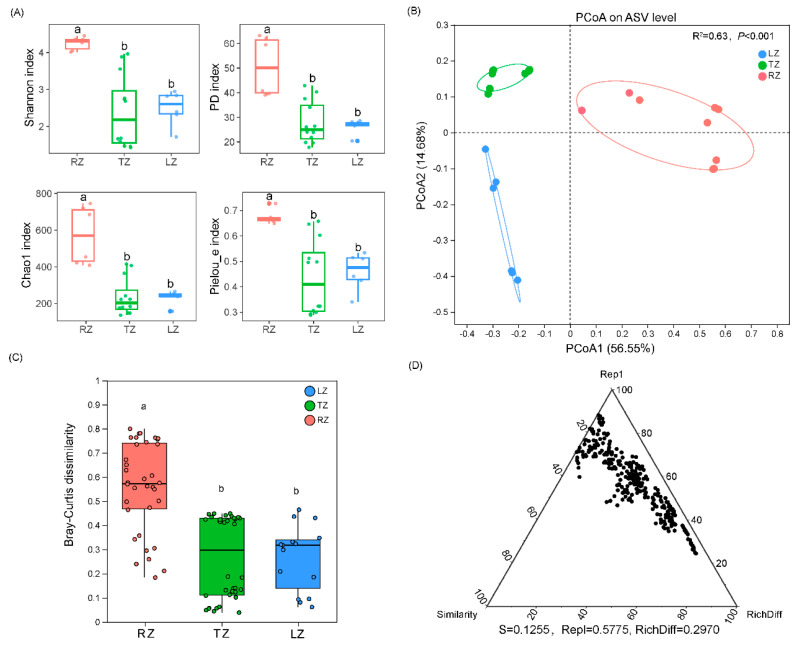
The diversity patterns of microeukaryotic plankton communities in different types of water areas. (**A**) Alpha diversity indices; (**B**) PCoA analysis based on the Bray–Curtis dissimilarity; (**C**) The community dissimilarity analysis; (**D**) The Partitioning Beta Diversity analysis of microeukaryotic plankton communities, similarity (S), species replacement (Repl), and richness difference (RichDiff). Different lowercase letters above the data boxes indicate significant difference (*p* < 0.05).

Analysis of α diversity indices revealed significant differences between RZ samples and the other two groups (*p* < 0.05) (Figure 2A). Specifically, the average Shannon index values were 4.09 for RZ, 1.94 for TZ, and 2.51 for LZ; the average PD index values were 47.23, 23.74, and 26.30, respectively; the average Chao1 index values were 514.07, 184.89, and 233.40, respectively; and the average Pielou evenness index values were 0.66, 0.37, and 0.46, respectively.

Principal coordinate analysis (PCoA) revealed that the first and second axes explained 56.55% and 14.68% of the total community variation across different zones, respectively (PERMANOVA R^2^ = 0.63, *p* = 0.001, Figure 2B). Community composition differed markedly between the RZ and the other two groups along the first axis, while the LZ and TZ were clearly separated along the second axis. Non-metric Multidimensional Scaling (NMDS) further revealed highly significant differences in community structure among the different zones of the Xiaolangdi Reservoir (R = 0.7466, *p* < 0.001; Figure S4A). Correlation analysis revealed a significant relationship between ASV richness and SPM concentration (Figure S2), ASV richness tended to increase with elevated SPM content, with this relationship explaining 85.7% of the total variance (*p* < 0.001). Bray–Curtis dissimilarity calculations revealed significant compositional differentiation between the RZ and the other two zones (*p* < 0.05) (Figure 2C). Results of β-diversity partitioning (species replacement and richness difference) revealed that the β-diversity of microeukaryotic communities was dominated by species turnover, which contributed 57.75%, whereas richness difference made a smaller contribution with 29.70% (Figure 2D). Among the three zones of species replacement contributed most to β-diversity in LZ (65.81%), followed by TZ (59.64%) and RZ (59.37%). By contrast, richness difference made the largest contribution in TZ (24.76%), then RZ (19.18%) and LZ (13.06%) (Figure S3). Furthermore, microeukaryotic plankton communities displayed a significant distance-decay pattern from the Yellow River mainstream to the Xiaolangdi Reservoir dam, with community similarity declining with increasing geographic distance (R^2^ = 0.581, *p* < 0.001; Figure S4B).

**Figure 3:**
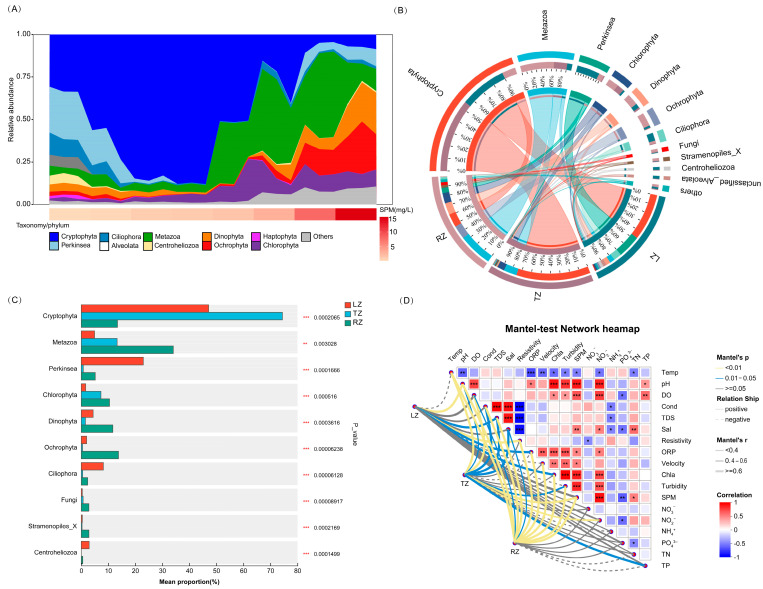
Shifts in microeukaryotic plankton community composition and key driving factors along the river-to-dam water gradient. (**A**) Dynamics of microeukaryotic plankton phylum-level composition in relation to SPM concentration; (**B**) Phylum-level relative abundance of dominant microeukaryotic plankton in three groups; (**C**) Compositional differentiation of microeukaryotic plankton among three groups; (**D**) Mantel test between microeukaryotic plankton diversity and environmental factors. Asterisks indicate statistical significance from two-sided Pearson correlation tests adjusted using the Benjamini–Hochberg method: * *p* < 0.05, ** *p* < 0.01, *** *p* < 0.001.

From the dam front to the riverine section, microeukaryotic plankton species composition exhibited significant shifts with increasing SPM concentrations (Figure 3A). Specifically, the dominant microeukaryotic plankton groups were Cryptophyta, Metazoa, Perkinsea, Chlorophyta, Dinophyta, Ochrophyta, Ciliophora, Fungi, Stramenopiles, and Centroheliozoa, accounting for 97.7% of the total abundance (Figure 3B). Distinct differences in the relative abundance of dominant taxa were observed among the three groups (Figure 3C). The relative abundances of Metazoa, Chlorophyta, Dinophyta, Ochrophyta, Stramenopiles_X, and Fungi were higher in the RZ. By contrast, Cryptophyta was more abundant in the TZ and LZ. Specifically, the TZ exhibited the highest relative abundances of Cryptophyta (74.41%) and Metazoa (13.27%). The RZ was dominated by Metazoa (34.05%), Chlorophyta (10.45%), Dinophyta (11.68%), Ochrophyta (13.78%), Stramenopiles_X (2.83%), and Fungi (2.82%). The LZ showed the highest relative abundances of Perkinsea (22.96%) and Ciliophora (8.18%). As revealed by correlation analysis between different water zones of the Xiaolangdi Reservoir and environmental variables (Figure 3D), SPM represented the key driving factor for both the RZ and TZ (r = 0.9327, *p* < 0.001; r = 0.9208, *p* < 0.001, respectively). Conversely, the LZ was mainly driven by TDS and ORP (r = 0.9094, *p* = 0.0167; r = 0.9068, *p* = 0.0083, respectively).

### 3.3. Spatial Assembly Mechanisms of Microeukaryotic Plankton Communities in the Xiaolangdi Reservoir

**Figure 4:**
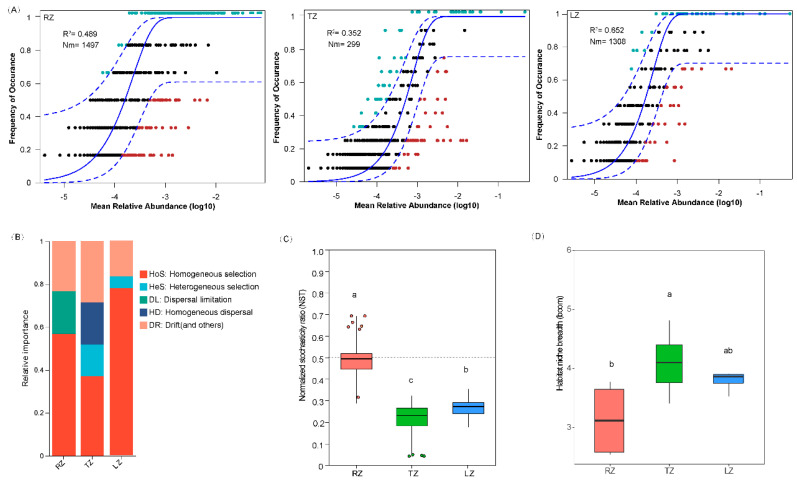
Deterministic and stochastic processes governing the assembly of microeukaryotic plankton community. (**A**) Fit of the neutral community model, the blue solid line shows the best fit of the neutral community model, and the blue dashed lines show the 95% confidence intervals (based on 1000 bootstrap replicates), ASVs with frequencies above or below the model predictions are color-coded accordingly; (**B**) Details the contributions of five distinct ecological processes to the microbial community; (**C**) Normalized stochastic ratio (NST), which is greater than 0.5 indicating dominance of stochastic processes and NST less than 0.5 indicating dominance of deterministic processes. (**D**) The habitat niche breadth. The letters denote statistically significant differences among groups (*p* < 0.05).

Based on the environmental differences among the three zones, we further explored the assembly mechanisms of microeukaryotic plankton communities. Neutral model analysis showed that the explanatory power (R^2^) of stochastic processes initially decreased and then increased along the SPM gradient, being lowest in the TZ (35.2%) and higher in the RZ (65.2%) and LZ (48.9%) (Figure 4A). This suggests that in the transitional zone—where environmental gradients are most pronounced—the relative contribution of stochastic processes is weakened, and deterministic processes become dominant.

Null model analysis further supported this conclusion. Homogeneous selection was the dominant deterministic process across all three zones, with the highest contribution in the RZ (77.78%), followed by the TZ (56.79%) and LZ (37.04%) (Figure 4B). Notably, as SPM concentration increased (i.e., from LZ to RZ), the overall contribution of deterministic processes increased. This is consistent with the normalized stochasticity ratio (NST) results: NST values were 48.87% in both the RZ and LZ, but only 21.82% in the TZ (Figure 4C). Taken together, these findings indicate that high sediment load strengthens environmental filtering, thereby promoting homogeneous selection as the dominant mechanism governing community assembly.

Interestingly, habitat niche breadth analysis revealed that the TZ exhibited the widest niche breadth (4.25), followed by the LZ (4.08), while the RZ showed the narrowest (3.20) (Figure 4D). This finding may seem counterintuitive—the zone with the most drastic environmental gradients supports the broadest niche breadth. One possible explanation is that the transitional zone, acting as an ecotone between the riverine and lacustrine zones, pools species from both upstream and downstream habitats. Simultaneously, the high heterogeneity of environmental factors in this zone provides opportunities for co-occurrence of taxa with different ecological preferences, thereby broadening the overall community niche breadth.

### 3.4. Spatial Variation in Co-Occurrence Networks of Microeukaryotic Plankton Community in the Xiaolangdi Reservoir

**Figure 5:**
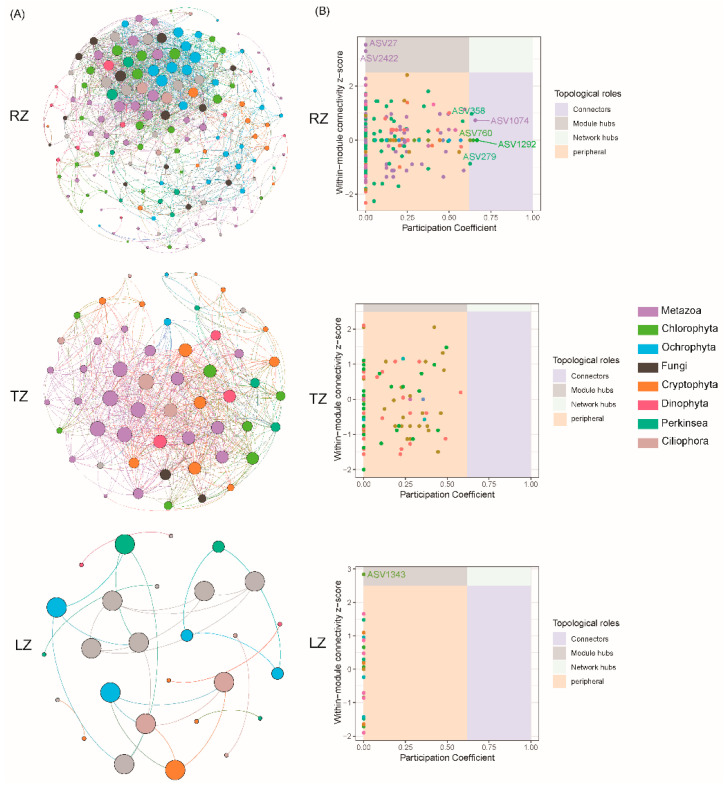
Co-occurrence network of microeukaryotic plankton communities in different zones of XiaoLangdi Reservoir. (**A**) The co-occurrence networks, the size of the circle indicated the relative abundance of the ASVs; (**B**) The key nodes in the networks are selected by within-module connectivity (Zi) and among-module connectivity (Pi).

In addition to being driven by abiotic factors, changes in microbial communities are also influenced by biotic interactions. To investigate biotic interactions within microeukaryotic plankton community in the Xiaolangdi Reservoir, we constructed co-occurrence networks across different water zones, revealing that network patterns varied with hydrological environments (Figure 5, Table S2). The networks across the three zones comprised 27–159 nodes linked by 27–1434 edges. The microeukaryotic plankton network in the RZ exhibited significantly higher node number, edge count, and average degree (159, 1434, and the corresponding value, respectively) than those in the other two zones, suggesting the highest network complexity in the RZ (Figure 5A). Compared with the other two zones, the TZ microeukaryotic plankton network exhibited the highest average degree, weighted coefficient, graph density, and clustering coefficient, but the lowest modularity and connected components, indicating stronger community connectivity, interactions, and more efficient transfer of information, energy, and materials (Figure 5A). Compared with those in the RZ and TZ, the LZ network showed lower graph density, higher modularity, shorter path length, and lower clustering coefficient, implying favorable local connectivity but weaker global connectivity and less efficient information transfer within the microeukaryotic plankton community (Figure 5A). Co-occurrence network topology revealed that interactions among microeukaryotic plankton in the Xiaolangdi Reservoir were dominated by positive correlations, with the highest proportion in the LZ (70.37%), followed by the TZ (65.77%) and the lowest in the RZ (59.14%), which indicated that microbial communities were mainly shaped by cooperative or mutualistic interactions (Table S2).

In the microeukaryotic plankton networks across the three zones, most nodes were peripheral, with only a few identified as module hubs and connectors and none classified as network hubs (Figure 5B, Table S3). In the RZ, two microeukaryotic plankton nodes were identified as module hubs, belonging to *Chrysochromulina* (Haptophyta) and Cryptomycotina_XX (Fungi), while five connector nodes were affiliated with *Melosira* (Ochrophyta), Novel-clade-2_X (*Melosira*), an unclassified Dinophyta genus, Hyceae_X (Metazoa), and *Eudiaptomus*_unclassified_f_Chlamydomonadales_X (Chlorophyta). No key taxa were detected in the TZ, and the RZ harbored the greatest number of key taxa. In the LZ, one unclassified genus affiliated with Centroheliozoa was recognized as a module hub. Among the eight key taxa identified, only *Chrysochromulina* (ASV27) in the RZ exhibited a relative abundance above 1%, with all others below 1%. Overall, these findings demonstrate that hydrological heterogeneity induced by dam construction decreases the resistance of riverine ecosystems to external perturbations and compromises their ecological resilience.

## 4. Discussion

### 4.1. Dynamic Shifts in the Composition and Diversity of Microeukaryotic Plankton Community in the Xiaolangdi Reservoir

Although numerous studies have investigated the spatiotemporal dynamics of the microeukaryotic plankton community in freshwater ecosystems [49,50], comparative analyses of their spatial dynamics in reservoirs associated with sediment-laden remain limited. For example, studies have shown that phytoplankton community composition in the Yellow River is mainly shaped by catchment erosion and tributary inputs, rather than limited by in situ algal growth [51]. High suspended sediment load attenuates the positive relationship between nitrogen nutrients and zooplankton diversity [52].

In this study, we examined microeukaryotic plankton communities across different zones of the Xiaolangdi Reservoir. Significant differences in community composition and diversity were observed among the riverine, transitional, and lacustrine zones (Figure 2 and Figure 3), which is consistent with a previous relevant study in the Yangtze River [53]. Dam construction converted the natural lotic river into a relatively lentic, lake-like system, restructuring the aquatic ecosystem [2] and driving shifts in the acclimatization strategies of aquatic organisms from lotic- to lentic-adapted assemblages.

Microeukaryotic diversity differed significantly among the three water types (Figure 2A). Alpha diversity was higher in the RZ than in the LZ, while beta diversity revealed strong community separation at both ASV and taxonomic levels (Figure 2B), with species turnover as the dominant component driving community compositional dynamics (Figure 2D and Figure S3). This finding is in agreement with previous studies, which demonstrated that high sediment load weakens zooplankton β-diversity through the reduction in habitat heterogeneity [52]. Environmental factors can largely explain the variations in eukaryotic plankton communities [54]. Our results revealed significant spatial heterogeneity in microeukaryotic plankton community composition across different zones of the Xiaolangdi Reservoir. Specifically, Cryptophyta dominated the LZ and TZ, while Ochrophyta was widely distributed and increased in relative abundance with decreasing SPM and higher water transparency. Previous studies reported that Ochrophyta gametophytes have strong photosynthetic plasticity, enabling adaptation to varying light, temperature, and pH conditions [55]. In contrast, Metazoa exhibited the highest abundance in riverine waters and correlated significantly with water temperature (Figure 3B,D). Water temperature predominantly drove species turnover in metazoan communities, reflecting their high physiological sensitivity to thermal gradients [56]. In this study, Cryptophyta showed higher relative abundance in areas with high turbidity and low water transparency, which is consistent with their low-light adaptation [57]. Chlorophyta, although widely distributed across the study area, was somewhat suppressed under extremely high sediment loads where light penetration was severely limited. This may reflect the limited light tolerance range of some Chlorophyta species, which cannot sustain growth under extremely low irradiance [58]. Together, these taxon-specific light adaptation characteristics provide important physiological and ecological explanations for the variation in phytoplankton community composition among different reaches of the Yellow River. This heterogeneity is primarily driven by variations in environmental conditions or ecological gradients [50]. Cryptophyta are generally considered a eurythermic group, capable of surviving across a wide temperature range, which explains their widespread distribution throughout the study area [59]. In contrast, certain Chlorophyta species may exhibit stenothermic characteristics, with their distribution restricted to a specific temperature range [60]. In addition, Plankton are widely used as sensitive indicators of aquatic ecosystem health due to their short life cycles and high environmental responsiveness [61]. Mantel test results demonstrated that the microeukaryotic plankton community in the TZ exhibited more significant correlations with environmental factors and stronger correlation coefficients than those in the LZ and RZ (Figure 3D). SPM acted as the key driver in the RZ and TZ, whereas TDS, electrical resistivity and ORP were the primary factors in the LZ, all of which were closely associated with water transparency, thereby promoting the richness and abundance of phytoplankton such as Ochrophyta in the reservoir (Figure 3A).

### 4.2. Assembly Mechanisms of Microeukaryotic Plankton Communities Across Distinct Water Zones

In this study, community assembly mechanism highlighted different roles in determining the composition of microeukaryotic plankton communities in different zones in Xiaolangdi Reservoir (Figure 4). We found that the microeukaryotic plankton community in the RZ was more strongly affected by dispersal limitation than in the LZ, likely due to the narrower niche breadth and dam effects in the RZ (Figure 4A,D). This finding is inconsistent with previous studies: in areas with high sediment load, homogeneous selection is enhanced while dispersal limitation is weakened in the assembly of algal communities [17]. This may be attributed to the fact that eukaryotic plankton communities include a wider array of taxonomic groups, not only phytoplankton but also other protists, fungi, and small metazoans. Under strong environmental selection, communities show weaker dispersal limitation, as environmental filtering leads to dominance by a few abundant species and large fluctuations in rare species [62]. Futhermore, the contribution of dispersal limitation to ecological processes was significantly higher in the RZ than in the LZ, further supporting this observation (Figure 4B). Higher river connectivity facilitates greater passive microbial dispersal and a higher immigration rate in the RZ, as reflected by its higher alpha diversity relative to the LZ (Figure 2A). Importantly, null model and NST analyses revealed that the assembly of microeukaryotic plankton communities in the Xiaolangdi Reservoir was primarily governed by deterministic processes. Although stochastic processes contributed more significantly to the riverine zone than to the lacustrine zone, homogeneous selection dominated both regions (Figure 4B,C). This pattern aligns with previous studies, highlighting the key role of homogeneous selection in shaping microbial community assembly [53].

Dams alter hydrological conditions and thereby shape microbial communities. Our study revealed that riverine SPM was a significant factor influencing microeukaryotic plankton in the RZ, with a stronger effect than in the LZ (Figure 3D). Moreover, Metazoa, Dinophyta, and Phaeophyta were enriched in high-SPM riverine waters, whereas Alveolata dominated low-SPM lacustrine zones (Figure 3A). Following reservoir impoundment, the shift from lotic to lentic hydrological regimes results in decreased flow velocity, elevated nutrient levels, and reduced habitat diversity in the river–reservoir ecosystem [63]. Apart from hydrological factors, SPM is an important influencing factor in river networks [64]. In conclusion, SPM strengthened the contribution of deterministic processes to microeukaryotic community assembly, whereas lotic conditions enhanced their dispersal ability. Nonetheless, distinct environmental selection pressures on species may result in discrepancies between neutral model predictions and empirical observations [65].

### 4.3. Spatial Variation in Complexity and Stability of Microeukaryotic Plankton Communities

Network analysis revealed that the riverine zone exhibited the highest network complexity and stability, characterized by more nodes, edges, and higher average degree, along with a more modular structure. This was unexpected, as high-turbidity environments might be expected to simplify biotic interactions. Several factors may contribute to this pattern. The interactions among microeukaryotes can reflect the biological interactions in ecosystems, where species are involved in complex positive (e.g., commensalism and mutualism) and negative (e.g., predation and competition) interactions [66]. The co-occurrence network relationships of microeukaryotes are regarded as important drivers of community assembly [67]. Although we cannot fully explain the biological interactions in microeukaryotic networks, these networks help us understand the complexity of communities and their responses to environmental changes [68]. Positive correlations represent parasitic relationships between communities, while negative correlations indicate predatory or competitive relationships between communities [34]. Co-occurrence network analysis showed that the network relationships in all three zones were dominated by positive correlations and exhibited modular structures (modularity coefficient ≥ 0.24), indicating that the relationships among species were mainly cooperative (Figure 5 and Table S2). Generally, networks with higher connectivity, complexity, and stability respond more rapidly to environmental disturbances [69].

We found that the RZ exhibited the highest network complexity, whereas the TZ showed stronger interconnection and closer associations among nodes (Figure 5A). The LZ exhibited the smallest network diameter and average path length (Table S2), suggesting tighter connectivity among microeukaryotic plankton communities in this zone. An excessively long average path length may elevate energy consumption required to maintain normal functioning, thereby adversely affecting ecosystem stability and function in this region [70]. Furthermore, the modularity of the LZ was higher than that of the other groups, indicating that the taxas in the LZ had stronger interconnectivity and closer connections between nodes. Such a highly modular structure enhances community stability by restricting the effects of species loss to individual modules and mitigating the propagation of extinction cascades across the entire network [71]. Within microeukaryotic networks, module hubs and connectors play critical roles in maintaining ecosystem stability and mediating community assembly processes [69]. These species play important structural roles in the network, and their disappearance may lead to the collapse of the entire network [72].

Evaluating the key factors affecting connectivity indices and identifying the key taxa of microeukaryotic networks can deepen our understanding of microeukaryotic community assembly [73]. Our results revealed that the RZ network contained 5 connectors and 2 module hubs, while the LZ network possessed only 1 module hub; no key taxa were identified in the TZ network (Figure 5B, Table S3). These results collectively demonstrate that the microeukaryotic plankton co-occurrence network in the RZ exhibited greater complexity and higher stability. The LZ community showed favorable local connectivity but weaker global connectivity and less efficient information transmission. In the TZ, plankton displayed stronger interspecific connectivity and interactions, as well as higher efficiency in information, energy, and material transfer, albeit with lower community stability.

From an ecological management perspective, the high network stability in the RZ implies that this zone may be more resilient to environmental perturbations, while the LZ, despite lower network complexity, might be more susceptible to regime shifts [74]. These findings provide scientific support for biodiversity conservation in large regulated rivers: maintaining hydrological connectivity and managing sediment inputs are critical for preserving the functional integrity of microeukaryotic communities across different reservoir zones.

### 4.4. Limitations of Metabarcoding-Based Taxonomic Classification

The taxonomic assignments in this study were generated using the SILVA database following a standard metabarcoding annotation workflow. While widely adopted in environmental sequencing research, several limitations inherent to this approach must be acknowledged. Firstly, the accuracy of taxonomic annotation is highly dependent on the completeness and taxonomic curation of the reference database. Although SILVA provides broad coverage across the eukaryotic tree of life, it exhibits known imbalances in sequence representation and taxonomic consistency, particularly for protistan lineages such as Dinophyta and Cryptophyta. Secondly, it should be noted that the annotation results generated by the SILVA database used in this study (e.g., “Metazoa”, “Dinophyta”, “Cryptophyta”) are operational taxonomic labels based on molecular sequence similarity, rather than formal taxonomic ranks as defined by the International Code of Zoological Nomenclature or the International Code of Nomenclature for algae, fungi, and plants. For instance, “Metazoa” in SILVA often appears as a high-level aggregate unit, whose taxonomic level (equivalent to “kingdom”) is not equivalent to that of “Dinophyta” (typically equivalent to “phylum”). Finally, size fractionation using a 200 μm filter cannot effectively remove the larvae, debris, and environmental DNA of large eukaryotic plankton. DNA high-throughput sequencing methods based on 200 μm size fractionation cannot avoid contamination from environmental DNA. Future quantitative studies will require integrated analyses combining RNA high-throughput sequencing and microscopy techniques [75]. Consequently, ecological interpretations based on such metabarcoding data should focus on relative abundance trends, functional guilds, or broader ecological strategies, rather than overemphasizing the absolute taxonomic identity of individual labels. Future studies may benefit from integrating alternative reference databases (e.g., PR2 for protists) or employing phylogenetic placement methods to cross-validate and refine taxonomic classifications.

## 5. Conclusions

This study analyzed microeukaryotic diversity, community assembly, and co-occurrence networks across the riverine (RZ), transitional (TZ), and lacustrine (LZ) zones of the Xiaolangdi Reservoir using 18S rRNA gene sequencing and environmental monitoring. The three zones differed significantly in environmental characteristics. RZ had the highest SPM (mean 8.73 mg/L), high flow velocity, and low transparency; LZ had the lowest SPM (mean 0.46 mg/L) and highest transparency; TZ was intermediate. Dominant taxa varied clearly among zones. High-SPM RZ was dominated by Metazoa, Dinophyta, and Ochrophyta; low-SPM LZ was dominated by Alveolata. SPM is the key driver of community differentiation. Anthropogenic damming had significant impacts. Compared to pre-dam conditions, community assembly shifted from stochastic- to deterministic-dominated processes (homogeneous selection), which increased along the SPM gradient. Ecological impacts were zone-specific. LZ showed increased transparency but reduced network connectivity; RZ maintained high diversity but high SPM stressed light-sensitive taxa. Damming altered natural successional patterns. The three zones now function as distinct ecological entities, with SPM as the primary environmental filter. Future management should adopt zone-specific strategies: sediment regulation in RZ, network vulnerability in LZ, and gradient-induced instability in TZ.

## Data Availability

All the raw data from this study was deposited in the National Center for Biotechnology Information (NCBI) Sequence Read Archive (SRA) under BioProject ID PRJNA1373512.

## Supplementary Materials

The following supporting information can be downloaded at: https://www.mdpi.com/article/10.3390/biology15120945/s1, Figure S1: Microeukaryotic plankton community rarefaction curves based on Sobs Index of amplicon sequence variants (ASVs) defined at 100% sequence similarity threshold; Figure S2: Shifts in the diversity of the microeukaryotic plankton community with the content of suspended particulate matter; Figure S3: The ternary map is used to compare the Partitioning Beta Diversity (Turnover and Nestedness Components) of microeukaryotic plankton communities in different types of water areas; Figure S4: Non-metric multidimensional scaling analysis and distance decay relationship of three types of water; Figure S5: The assembly mechanism of microeukaryotic plankton communities; Table S1: Environmental factors in different aquatic zones during the survey period; Table S2: Topological characteristics of the community network of microeukaryotic plankton; Table S3: Taxonomic information of keystone taxa in eukaryotic plankton co-occurrence networks across different types of water areas in XiaoLangdi Reservoir.
