# Supplementary material for: Community Composition, Assembly Processes and Stability of Microeukaryotic Plankton in Response to Damming-Altered Heterogeneous Hydrology in a Sediment-Laden River
Source: Biology (Basel). 2026 Jun 17;15(12):945. doi: 10.3390/biology15120945 (PMC13295451; doi:10.3390/biology15120945)
Supplement: Supplementary file 1 [file biology-15-00945-s001.zip › biology-4259830-supplementary.pdf]

Community Composition, Assembly Processes and Stability of Microeukaryotic Plankton in Response to Damming-Altered Heterogeneous Hydrology in a Sediment-Laden River

Huatao Yuan[a,b,c], Junjun Mei[a], Xucong Lyu[a], Xiaofei Gao[a,b,c], Jing Dong[a,b,c], Jingxiao Zhang[a,b,c], Penghui Zhu[a,b,c], Yunni Gao[a,b,c*], Xuejun Li[a,b,c*]

[a]*College of Fisheries, Henan Normal University, Xinxiang, Henan, 453007, China*

[b]*Observation and Research Station on Water Ecosystem in Danjiangkou Reservoir of Henan Province, Nanyang, Henan, 474450, China*

[c]*The National Ecological Quality Comprehensive Monitoring Station (Hebi Station), Hebi, 458000, China*

***Corresponding authors at**: College of Fisheries, Henan Normal University, Xinxiang 453007, China.

Email address: gaoyn@htu.cn(Y. Gao), xjli@htu.cn (X. Li).

Table S1 Environmental factors in different aquatic zones during the survey period

	RZ	TZ	LZ
Temp	22.8444±1.4293^{a}	23.7556±0.5769^{a}	23.9833±0.3131^{a}
pH	8.1389±0.0362^{a}	7.9667±0.0958^{b}	7.8933±0.0407^{b}
DO	11.6933±0.1315^{a}	10.0567±0.4422^{b}	10.4450±0.5706^{b}
Cond	1093.0000±10.8972^{b}	1113.7778±10.6628^{a}	1076.5000±6.2383^{c}
TDS	540.3333±4.4441^{a}	550.1111±6.1328^{a}	532.3333±3.3500^{b}
Sal	0.5467±0.0050^{a}	0.5500±0.0087^{a}	0.5333±0.0047^{b}
Resistivity	915.5556±11.0353^{ab}	901.8889±11.7627^{b}	927.3333±6.3944^{a}
ORP	230.2222±31.0775^{a}	211.6000±3.7570^{a}	201.7833±10.8304^{a}
Velocity	0.6549±0.0672^{a}	0.3234±0.0393^{b}	0.0688±0.0086^{c}
Chl a	3.4406±1.7938^{a}	1.0593±0.1314^{b}	0.5688±0.0759^{c}
Turbidity	3.4070±1.5452^{a}	1.5309±0.4118^{b}	0.9118±0.1209^{c}
SPM	8.7333±5.1391^{a}	1.4667±0.2646^{b}	0.5000±0.1000^{c}
NO_3	3.3555±0.0737^{a}	3.3420±0.1745^{a}	3.3686±0.0509^{a}
NO_2	0.0439±0.0032^{a}	0.0336±0.0044^{b}	0.0265±0.0020^{c}
NH_4	0.0306±0.0041^{a}	0.0281±0.0142^{a}	0.0458±0.0153^{b}
PO_4	0.0108±0.0035^{b}	0.0210±0.0171^{ab}	0.0196±0.0047^{a}
TN	4.0166±0.1376^{a}	3.9892±0.1652^{a}	3.8759±0.0608^{a}
TP	0.0278±0.0028^{a}	0.0244±0.0026^{a}	0.0274±0.0047^{a}

Different lowercase letters indicate significant differences within different types of water body groups($P < 0.05$).

Table S2 Topological characteristics of the community network of microeukaryotic plankton

Network indexs	RZ	TZ	LZ
Number of Nodes	159	54	27
Edges	1434	593	27
Connected Component	3	1	10
Network Diameter	8	6	1
Average Degree	18.04	21.96	2
Avg. Weighted Degree	16.08	18.48	1.99
Modularity	0.37	0.24	0.83
Graph Density	0.11	0.41	0.08
Avg. Path Length	2.96	1.83	1
Avg.Clustering Coefficient	0.58	0.79	0
Positive correlation	59.14%	65.77%	70.37%
Negative correlation	40.86%	34.23%	29.63%

Table S3 Taxonomic information of keystone taxa in eukaryotic plankton co-occurrence networks across different types of water areas in XiaoLangdi Reservoir

Group	Node type	ASVs	Relative abundance	Classification	
				Phylum	Genus
RZ	Module hubs	ASV27<br>ASV2422	0.1130 %<br>0.0189 %	Haptophyta<br>Fungi	Chrysochromulina<br>Cryptomycotina_X<br>X
	Network hubs	NA	NA	NA	NA
	Connectors	ASV358<br>ASV1074<br>ASV760<br>ASV1292<br>ASV279	0.0246%<br>0.011%<br>0.0048%<br>0.0408%<br>0.0222%	Ochrophyta<br>Melosira<br>Dinophyta<br>Metazoa<br>Chlorophyta	Melosira<br>Novel-clade-2_X<br>unclassified_o_Dino<br>phyceae_X<br>Eudiaptomus<br>unclassified_f_Chla<br>mydomonadales_X
TZ	Module hubs	NA	NA	NA	NA
	Network hubs	NA	NA	NA	NA
	Connectors	NA	NA	NA	NA
LZ	Module hubs	ASV1343	0.001%	Centroheliozo<br>a	unclassified_c__Cen<br>troheliozoa_X
	Network hubs	NA	NA	NA	NA
	Connectors	NA	NA	NA	NA

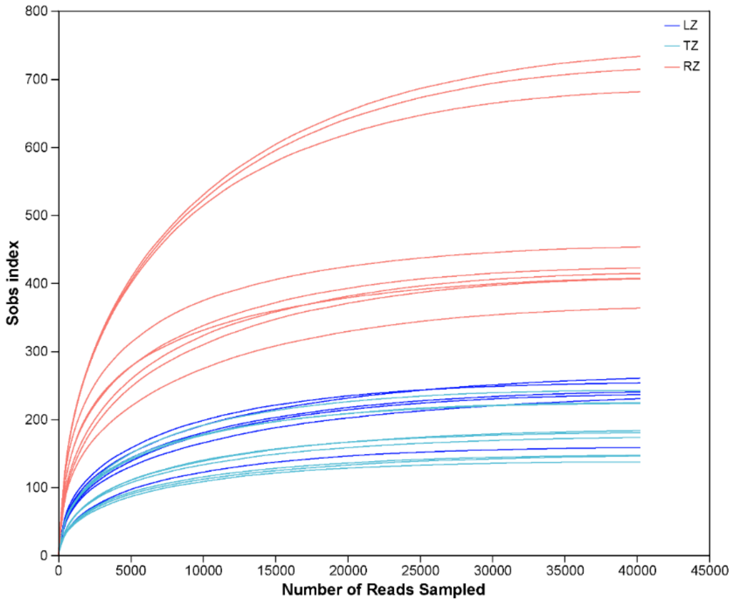

Figure S1. Microeukaryotic plankton community rarefaction curves based on Sobs Index of amplicon sequence variants (ASVs) defined at 100% sequence similarity threshold.

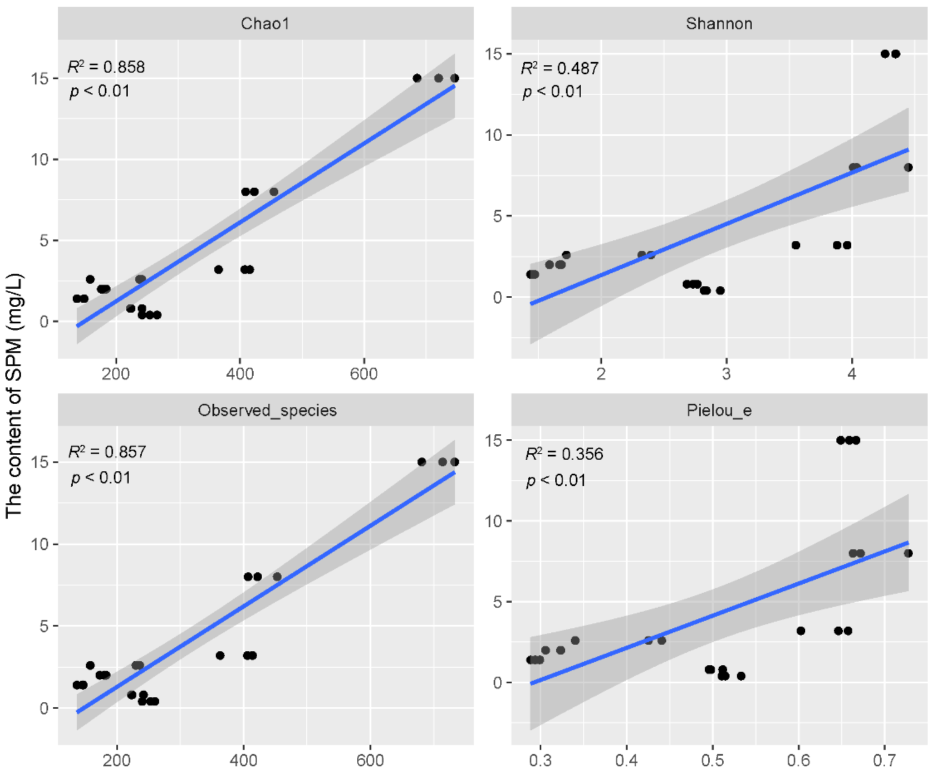

Figure S2. Shifts in the diversity of the microeukaryotic plankton community with the content of suspended particulate matter.

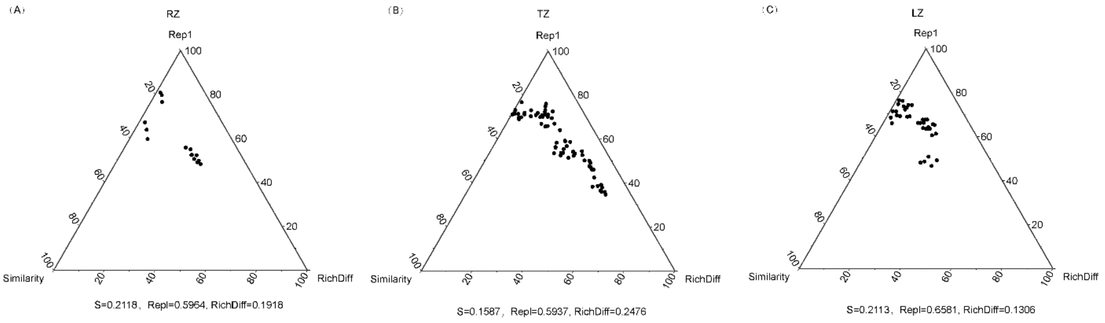

Figure S3. The ternary map is used to compare the Partitioning Beta Diversity(Turnover and Nestedness Components) of microeukaryotic plankton communities in different types of water areas. (A)RZ;(B)TZ;(C)LZ. Each point in the diagram represents a pair of sites, whose positions are determined by the mean values derived from three matrices: similarity (S), species replacement (Repl), and richness difference (RichDiff). Each corresponding triple of values sums to one.

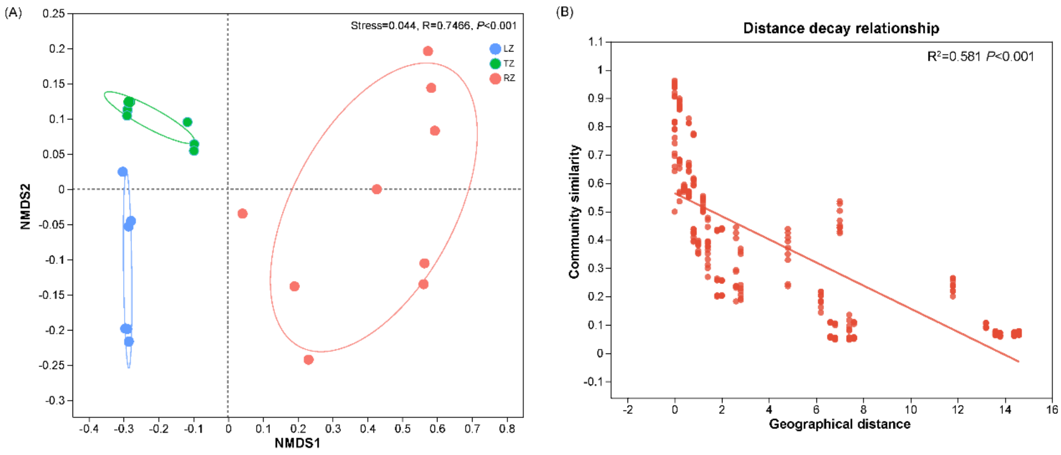

Figure S4. Non-metric multidimensional scaling analysis and distance decay relationship of three types of water.

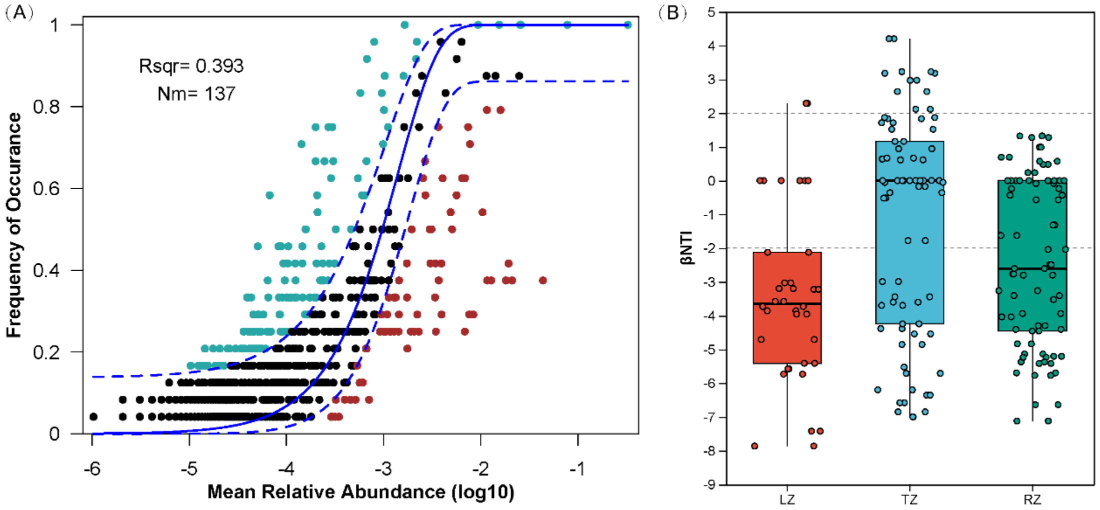

Figure S5. The assembly mechanism of microeukaryotic plankton communities.
